# Histidine 352 (His^352^) and Tryptophan 355 (Trp^355^) Are Essential for Flax UGT74S1 Glucosylation Activity toward Secoisolariciresinol

**DOI:** 10.1371/journal.pone.0116248

**Published:** 2015-02-25

**Authors:** Kaushik Ghose, Jason McCallum, Marva Sweeney-Nixon, Bourlaye Fofana

**Affiliations:** 1 Crops and Livestock Research Centre, Agriculture and Agri-Food Canada, 440 University Avenue, Charlottetown, Prince Edward Island, C1A 4N6, Canada; 2 University of Prince Edward Island, 550 University Avenue, Charlottetown, Prince Edward Island, C1A 4P3, Canada; University of Manitoba, Canada

## Abstract

Flax secoisolariciresinol diglucoside (SDG) lignan is a natural phytoestrogen for which a positive role in metabolic diseases is emerging. Until recently however, much less was known about SDG and its monoglucoside (SMG) biosynthesis. Lately, flax UGT74S1 was identified and characterized as an enzyme sequentially glucosylating secoisolariciresinol (SECO) into SMG and SDG when expressed in yeast. However, the amino acids critical for UGT74S1 glucosyltransferase activity were unknown. A 3D structural modeling and docking, site-directed mutagenesis of five amino acids in the plant secondary product glycosyltransferase (PSPG) motif, and enzyme assays were conducted. UGT74S1 appeared to be structurally similar to the *Arabidopsis thaliana* UGT72B1 model. The ligand docking predicted Ser^357^ and Trp^355^ as binding to the phosphate and hydroxyl groups of UDP-glucose, whereas Cys^335^, Gln^337^ and Trp^355^ were predicted to bind the 7-OH, 2-OCH_3_ and 17-OCH_3_ of SECO. Site-directed mutagenesis of Cys^335^, Gln^337^, His^352^, Trp^355^ and Ser^357^_,_ and enzyme assays revealed an alteration of these binding sites and a significant reduction of UGT74S1 glucosyltransferase catalytic activity towards SECO and UDP-glucose in all mutants. A complete abolition of UGT74S1 activity was observed when Trp^355^ was substituted to Ala^355^ and Gly^355^ or when changing His^352^ to Asp^352^_,_ and an altered metabolite profile was observed in *Cys335Ala*, *Gln337Ala*, and *Ser357Ala* mutants. This study provided for the first time evidence that Trp^355^ and His^352^ are critical for UGT74S1’s glucosylation activity toward SECO and suggested the possibility for SMG production *in vitro*.

## Introduction

Lignans are a class of diphenolic nonsteroidal phytoestrogens with a wide variety of purported health benefits [1]–[8]. Different types of lignans have been reported in various plant species and include secoisolariciresinol diglucoside (SDG) encountered mainly in flax (*Linum usitatissimum* L.) seed [9]–[14]. Flax lignans are usually found glycosylated in oligomeric chains [15]; its aglycone (SECO, MW  = 362.4 g/mol) and monoglucoside (SMG) forms not being naturally accumulated *in planta*. Recently, During et al. [16] reported a linearly increased uptake of lignan aglycone forms (pinolariciresinol – PINO, SECO, and enterolactone – ENL) by human intestinal Caco-2 cells through simple diffusion or by low affinity transporter. Only <0.1% SDG uptake was observed compared to 2% SECO, 2% PINO, and 7% ENL uptake by Caco-2 cells, evidencing the effect of glucosylation on absorption and bioavailability [16]. Due to its lower molecular weight (MW  = 524.6 g/mol), it is reasonable to anticipate that SMG may be more prone to uptake through diffusion by Caco-2 cells compared to SDG (MW  = 686.7 g/mol). *In planta*, glycosylation is a key mechanism that determines the chemical complexity and diversity of plant natural products [17], [18], ensuring their chemical stability and water solubility while reducing chemical reactivity or toxicity [19], and facilitating their sorting, intercellular transport, storage and accumulation in plant cells [20]–[21]. Glycosylation is catalyzed by carbohydrate active enzymes (CAZymes), which include the glycosyltransferase (GT) superfamily [22]. Members of the GT superfamily have been classified into 94 families, with family 1 referred to as uridine glycosyl transferases (UGTs) [23], [24]. Plant UGTs are characterized by a 44 amino acid signature motif known as plant secondary product glycosyltransferase (PSPG) box [15], [24], [25]. UGTs transfer UDP-activated sugar moieties, including UDP-glucose, to specific acceptor molecules [26] and contribute to their structural diversity. Based on sequence homology, more than 120 UGTs have been reported in *Arabidopsis* and were grouped into 30 sub-families, classified from UGT71 to UGT100 [22]. Recently, Barvkar et al. [27] reported 137 functionally uncharacterized flax UGTs from the flax draft genome [28]. Concomitantly to Barvkar’s study [27], we have cloned and characterized five family 1 UGT genes belonging to four UGT families and to five sub-families referred to as *UGT74S1*, *UGT74T1*, *UGT89B3*, *UGT94H1*, *UGT712B1*. The functional characterization of the five UGTs identified UGT74S1 as the only one using SECO as substrate, forming SECO monoglucoside (SMG) and then SDG in a sequential manner [29]. Nonetheless, its catalytic mechanisms were not known. Many plant UGTs have been shown to be more regiospecific than substrate specific [30], [31]. In flax, UGT74S1 was shown to glucosylate only SECO among the different aglycones tested [29]. However, its strict substrate specificity is unknown and its regiospecificity cannot be ruled out.

Despite the large plant UGT sequence data available in databases, the crystal structures of only a few have been reported so far [36]. The gap between the number of existing sequences and structures has driven computational methods for predicting protein structures [32]. Homology modeling and structure-based protein–ligand molecular interaction docking analyses have become powerful tools for predicting functional residues in proteins, guiding the development of functional hypotheses [32]–[35], rationalizing experimental data, and designing directed mutagenesis experiments [37]. As such, 3D structure modeling and docking of UGT85B1 and UGT94B1 proteins with ligands have been reported in several plant species including *Sorghum bicolor* and *Bellis perennis* [38], [39]. Whereas the general role for the PSPG motif in substrate recognition and catalytic activity is widely accepted [18], [39], the specific role for individual amino acids within this motif is still a matter of debate and extensive investigations. Structure-based rational mutagenesis studies have helped in the identification of key amino acids involved in substrate binding and catalysis of UGTs, and have generated mutants with altered regiospecificity, compromised activity or turnover [41]. In *Medicago truncatula*, Phe^148^ and Tyr^202^ were found to control regiospecificity for quercetin glucosylation by UGT71G1 [30]. In soybean, Glu^392^ was identified as critical for GmlF7GT primary catalysis [42]. Currently, little is known about the key amino acids playing an essential role in the flax UGT74S1 glucosylation activity toward SECO. The objective of this study was to determine the role played by 5 amino acids located within the PSPG motif of the flax UGT74S1 in SECO glucosylation. Using 3D structural modeling of UGT74S1 protein, ligand docking, site-directed mutagenesis, heterologous expression and enzyme assays, we showed that Gln^337^ and Ser^357^ are essential for SMG conversion to SDG and that Trp^355^ and His^352^ are key critical amino acids within the PSPG motif and are determinant for UGT74S1 glucosylation activity toward SECO *in vitro.*

## Materials and Methods

### Molecular modeling and docking

Secondary and 3D protein structures were predicted using Protein Homology/analogy Recognition Engine V2.0 (Phyre2) software [32]. Briefly, the hidden Markov model (HMM) of UGT74S1 (JX011632; AGD95005) was constructed by iteratively detecting homologs in a protein database using PSI blast search engine. These homologs were scanned against experimentally solved structures present in an HMM protein library**.** The 3D protein models were constructed based on the alignments between the HMM of UGT74S1 sequence and the HMMs of known structures. The UGT74S1 structure was finally determined using the structure of the *Arabidopsis thaliana* UGT72B1 (Q9M156; PDB ID, d2vcha1; X-RAY DIFFRACTION with resolution of 1.45 Å) [43] as the closest search model. The generated protein model of wild type UGT74S1 was then subjected to beta testing using Phyre2 Investigator [32], interactively examining the pocket detection. The ZINC database codes [44] for three different conformers of SECO (2020114, 14694365, 14694366) and one for UDP-glucose (30320665), were used individually with the designed 3D protein structure of UGT74S1 for docking in SwissDock [34]. The predicted binding sites between each SECO structure or UDP-glucose and the UGT74S1 protein were clustered and visualized using UCSF Chimera [45]. Only the clusters and models showing the lowest energy and highest number of inter-molecular H bonds were selected, using default parameters (2 Å bond length and 0.4 Å relaxation), and applied for the rest of the study in docking and ligand binding sites predictions in the mutant variants.

### Site-directed mutagenesis

Cloning of the full length *UGT74S1* cDNA and its protein expression have been previously described [29]. The pYES2/NT C plasmid constructs harboring the full length cDNA for *UGT74S1* was used as a template for all the site-directed mutagenesis reactions. Amino acids targeted for mutation were those predicted as binding the sugar donor or the acceptor to the wild UGT74S1 in molecular docking analysis. The highly conserved His^352^ was also included as a target. These amino acids were substituted with smaller hydrophobic amino acids such as alanine, glycine, or the charged aspartic acid based on Phyre2 Investigator server prediction. This server predicts the effects of amino acid substitutions using the SuSPect method, a standalone web server that generates a mutational graph analysis by modelling the effect of specific amino acid mutations within the UGT74S1 protein sequence. Gene-specific primers containing the desired mutations were designed in such a way that they were 100% complementary to one another, had no overhangs, and the mutation site was centrally located on both primers (Table S1). The sequences of the wild UGT74S1 used for designing the mutagenesis forward and reverse primers are presented in S1 Fig. These primer sets were used to mutate 5 targeted amino acids by performing a DNA methylation and mutagenesis reaction as instructed by the GENart site-directed mutagenesis system (Invitrogen, Carlsbad, CA, USA). Briefly, the final methylation and mutagenesis reaction mixture consisted of 5 µl of 10X AccuPrime *Pfx* reaction mix, 5 µl of 10X Enhancer, 1.5 µl of primer mix (10 µM each), 20 ng of plasmid DNA, 1.5 µl of DNA Methylase (4 U/µL), 2.0 µl of 25X SAM, 0.4 µl of AccuPrime *Pfx* taq (2.5 U/µL), and PCR grade water (Life Technologies, Carlsbad, CA, USA), for a final volume of 50 µL. The methylation reactions were activated at 37°C for 20 min and the inverted mutagenesis PCR cycling conditions consisted of an initial denaturation at 94°C for 2 min followed by 18 cycles of 94°C for 20 s, 57°C for 30 s, and 68°C for 3 min. The final extension was carried out at 68°C for 5 min. After the reactions, 5 µL of the PCR products were visualized on a 0.8% agarose gel and the remaining products were purified using a PCR purification kit (Qiagen, Hilden, Germany).

### In vitro DNA recombination and bacterial transformation

The *in vitro* DNA recombination reaction mixture consisted of 4 µL of 5× reaction buffer, 2 µL of 10× Enzymer mix (Invitrogen), 8 µL of purified PCR product from the mutagenesis reaction, and 6 µL of PCR grade water, for a final volume of 20 µL. The mixture was incubated at room temperature for 10 min and the recombination reaction was stopped by adding 1 µL of 0.5 M EDTA prior to transformation into *E. coli* strain DH5α-T1R. A 5 µL of recombination product and 50 µL DH5α-T1R cells were used for the transformation. A total of 100 µL from 10-fold diluted transformation reaction was spread on pre-warmed LB agar plates containing 50 µg/mL of ampicillin and incubated at 37°C for 16–20 h. From each of the six transformation events, 10 colonies were analyzed by colony PCR using the *UGT74S1* gene-specific forward and reverse primers as well as the pYES2/NT C plasmid vector-specific T7 forward and CYC reverse primers. Plasmid DNA from three positive clones of each transformation event was sequenced by the Sanger method using the same UGT74S1 gene-specific and pYES2/NT C vector-specific primer pairs for confirmation of mutations.

### Heterologous expression

The pYES2/NT C plasmid constructs harbouring the cDNA of wild type *UGT74S1* and those of the 6 mutant *UGT74S1* genes were used to transform yeast INVSc1 strains, as described by Ghose et al. [29]. Briefly, single transformant INVSc1 yeast colonies were inoculated into 15 mL of *Saccharomyces cerevisiae* minimal media without uracil (SC-U), prepared as recommended by the supplier (Invitrogen), supplemented with 2% raffinose, and grown for 3 days under shaking at 250 *rpm* at 30°C until the OD_600_ reached 2.0. The culture was diluted in 50 mL of induction medium (SC-U supplemented with 1% raffinose and 2% galactose) to achieve an initial OD_600_ of 0.4 and further incubated under shaking at 30°C for 8 h. The induced yeast cells were harvested by centrifugation at 1,500 *g* for 5 min at 4°C. The cells were washed using 500 µL of sterile cold distilled water, centrifuged and the pellets washed with 500 µL of lysis buffer (50 mM sodium phosphate, pH 7.4 supplemented with 5% glycerol and 1 mM PMSF) at 4°C. After centrifugation, the cells were mechanically disrupted by vortexing for 30 seconds in the presence of an equal volume of 425–600 µm acid-washed glass beads (Sigma-Aldrich, St. Louis, MO, USA) and incubated on ice for 30 s. The vortexing and incubation cycle was repeated 4 times to ensure complete cell lysis. The lysates were centrifuged at 18,620 *g* for 10 min at 4°C and the supernatant was collected. The polyhistidine containing recombinant proteins were purified using the ProBond (Invitrogen) purification system following the manufacturer’s instructions. The purified enzymes were concentrated using 0.5 mL Ultracel-10k Amicon membrane column (Millipore, Billerica, MS, USA). Protein concentration was determined using the Bradford protein assay kit (Bio-Rad Laboratories, Hercules, CA, USA). The stability and expression of all six mutant proteins were monitored by Western blotting in which 40 µg total protein was separated on 12% polyacrylamide gel and transferred onto Immuno-blot PVDF membranes (Bio-Rad) using the Trans-Blot SD semi dry Transfer System (Bio-Rad). The membrane was first blocked in 5% nonfat dry milk blotting grade blocker (Bio-Rad) in TBST and incubated overnight with goat Anti-Xpress monoclonal antibody (1∶5,000 dilution, Life Technologies). The blots were washed with 0.05% TBST and incubated for one hour with HRP conjugated rabbit anti goat IgG secondary antibody (1∶6,000 dilution, Bio-Rad). After washing with 0.05% TBST, protein bands were developed using Immunostar Western C chemiluminescence kit western Blotting Detection Reagent (Bio-Rad) and photographed with a ChemiDocXRS molecular imaging system (Bio-Rad).

### Enzyme assays and UPLC metabolite profiling

The purified recombinant proteins obtained from the yeast cultures harboring the wild type or the six mutant variants of UGT74S1 were reacted with acceptor substrate SECO (Chromadex, Irvine, CA, USA), in the presence of UDP-glucose following the optimized conditions reported in [29]. Other alternative aglycones including quercetin, kaempferol, coumaric acid, and caffeic acid were also tested as substrates with each mutant and wild type of UGT74S1. The 100 µL reaction mixture consisted of a reaction buffer (50 mM sodium phosphate, 1 mM PMSF, 5% glycerol, pH 7.4), 280 µM aglycone SECO substrate (acceptor for glucosylation), and 1.64 mM UDP-glucose (sugar donor) (Sigma-Aldrich). The reaction mixtures were pre-incubated at 30°C for 10 min and the reactions were initiated by addition of 80 µg of enzyme. After incubation at 30°C for 30 min, the reactions were stopped with 100 µL of 0.5% trifluoroacetic acid in acetonitrile. The reaction mixtures were purified using 0.2 µm membrane filters (Pall Life Sciences, Port Washington, NY, USA) to remove any particulates that might form during the reaction. The separation and identification of the reactants and resulting products were carried out using a Waters H-Class Acquity UPLC system (Waters, Milford, MA, USA) equipped with a TQD tandem mass spectrometer (Waters) using a Waters CSH C18 column (100 mm×2.1 mm, 1.8 µm particle size). The formation of glucosylated products was monitored by examining the parent *m/z* masses and the principle fragments of eluted peaks, via ESI–mass spectrometry [29]. Two parallel MS2 scans were performed ranging from 120–800 a.m.u., using 15 and 45 V cone voltages. Selected ion recording (SIR) spectra were also collected to enhance the sensitivity of detection for SECO, SMG and SDG. The capillary voltage was set to 3 kV, the extractor and RF lens at 0.1 V. Chromatographic conditions consisted of a ternary gradient system composed of water (A), acetonitrile (B), and 10% formic acid in water (C), varied according to the following gradient: t0, A  = 68%, B  = 2%, C  = 30%; t1 = 4.4 min, A  = 0%, B  = 70%, C  = 30%; held isocratically until 6 min, afterward ramped down to starting conditions at 7 min, then held isocratically for 1 min to equilibrate before the next injection. Peaks were detected at 280 nm, indicative of phenolic lignan compounds, and were validated using authentic standards (SECO and SDG) purchased from Chromadex (Chromadex, Irvine, CA, USA). An in house standard of SMG was prepared by partial acid hydrolysis of SDG, purified and validated by LC-MS and NMR; SDG itself being generated and purified by alkaline hydrolysis of bulk flaxseed lignans [29]. All the reactions were carried out in three replicates and the data presented are the means ± standard deviations. A one-tailed student’s t-test was performed to test metabolite production levels by UGT74S1 variants.

### Wild and mutant UGT74S1 kinetic parameters

The kinetic parameters of the wild type and two UGT74S1 mutants were determined using a range of concentrations for sugar acceptor substrate (70–1650 µM SECO with fixed 1.64 mM UDP-glucose) or a range of concentrations for sugar donor (0.82–8.2 mM UDP-glucose with fixed 280 µM SECO) for 30 min, under optimum conditions described by Ghose et al. [29]. A total of 80 µg protein was used to determine the apparent *V*max and *K*m values for SECO and UDP-glucose the from the Lineweaver-Burk plots. The *k*cat was determined by dividing *V*max by the molar concentration of the enzyme.

## Results

### Modeling and molecular docking of wild type UGT74S1 with UDP-glucose and SECO

To determine key catalytic amino acids involved in UGT74S1 glucosylation mechanisms, 3D molecular modeling and ligand docking were conducted. By modeling wild type UGT74S1 against the PDB molecule UDP-glucosyltransferase (PDB ID, d2vcha1, Q9M156) using Phyre 2 tools, 458 amino acid residues, representing 98% of the total amino acid sequence, were successfully modeled with more than 90% accuracy. The generated 3D structure (S2 Fig.) had 1.45Å resolution with 100% confidence. The PSI Blast search showed a 25% identity between the wild UGT74S1 and the PDB target molecule. Phyre2 investigator 2D prediction detected the amino acids potentially located in the enzyme pocket, as well as amino acids with highest mutational sensitivity, mainly found clustered within the PSPG region (S3 Fig.).

**Figure 1:**
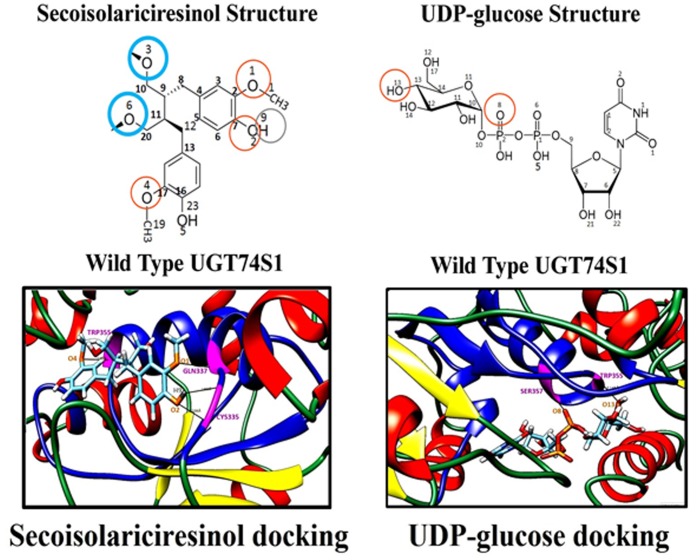
Molecular docking of wild type UGT74S1 with SECO and UDP glucose used as ligands. The structure of ligand SECO and UDP-glucose are presented on top of the docking models, where atoms are numbered following the UCSF Chimera numbering system (which is different from standard IUPAC numbering system). Only the portion of the protein interacting with ligands is shown. The α-helix, β-strand, and coils are colored in red, yellow green, respectively; the hydrogen bonds between amino acids and ligands and their respective length are indicated in black. The PSPG region is indicated in blue; amino acid residues involved in binding the sugar donor UDP-glucose or sugar acceptor SECO are colored in pink; the ligand oxygen atoms involved in hydrogen bond formation are circled in orange; the ligand hydrogen atoms involved in hydrogen bond formation are circled in grey; the SECO ligand oxygen atoms targeted for glucosylation are circled in blue.

Using the UGT74S1’s 3D structure and its ligand (SECO or UDP-glucose) ZINC codes, the enzyme-ligand binding sites were predicted (Table 1; Fig. 1). Two hydrogen bonds were found to be involved in binding UDP-glucose to the UGT74S1 protein. The first bond occurred between the serine at position 357 (Ser^357^) and the oxygen atom 8 located on the phosphate group 2 within the UDP-glucose moiety, forming a 2.207 Å hydrogen bond (Table 1; Fig. 1). The second bond occurred between the tryptophan at position 355 (Trp^355^) and the oxygen atom 13 of the hydroxyl group located at position 13 of UDP-glucose, forming a 2.145 Å hydrogen bond (Table 1; Fig. 1). These UDP-glucose binding sites were located within the HCGWNS region of PSPG. Similarly, the docking analysis predicted three amino acid residues to be involved in four hydrogen bond formations with SECO. First, the oxygen atom 1 of the methoxy-ester group at position 2 of SECO formed a 2.512 Å bond with the glutamine at position 337 (Gln^337^). Secondly, the hydrogen 9 of the hydroxyl group located at position 7 of the SECO formed a 1.802 Å bond with cysteine at position 335 (Cys^335^). Thirdly, the oxygen atom 2 of the hydroxyl group at position 7 of the SECO formed a 2.566 Å bond with cysteine at position 335 (Cys^335^), and fourthly, the oxygen atom 4 of the methoxy-ester group at position 17 of SECO formed a 2.486 Å bond with tryptophan at position 355 (Trp^355^) of UGT74S1 (Table 1, Fig. 1). Based on Phyre2 investigator and molecular docking data, Cys^335^, Gln^337^, Trp^355^, Ser^357^ were selected together with His^352^ for site-directed mutagenesis.

**Table 1:** Predicted binding site characteristics for the wild type UGT74S1 with its ligands UDP-glucose and SECO.

Protein	Ligand	Zinc code	Number of hydrogen bonds	Amino acid binding position	Binding functional group in ligand†	Bond size (Å)
UGT74S1	UDP-Glucose	30320665	2	Ser357	O8	2.207
				Trp355	O13	2.145
UGT74S1	SECO	14694366	4	Cys335	O2	2.566
				Cys335	H9	1.802
				Gln337	O1	2.512
				Trp355	O4	2.486

† Atoms were numbered following the UCSF Chimera numbering system (which is different from standard chemistry numbering system).

### In vitro site-directed mutagenesis

Using 20 possible amino acids within the query protein (wild type UGT74S1), SusPect method predicted the effect of their mutation in impairing the enzyme function. Substitutions of Cys^335^, Gln^337^, Trp^355^, Ser^357^, and His^352^ by Ala, Gly or Asp were predicted to have high likelihood to impair UGT74S1 function (S4 Fig.), thus giving more power to the selection of the targeted amino acids based on docking data. Using six pairs of primers targeting the 5 amino acids in site-directed mutagenesis, 6 mutated full length cDNA sequences were generated and confirmed by sequencing (S5 Fig.). These 6 mutated cDNAs were translated *in silico* into 6 mutant proteins referred to as Cys335Ala, Gln337Ala, Ser357Ala, Trp355Ala, Trp355Gly, and His352Asp mutants.

### Comparative 2D structures of the wild and mutant UGT74S1 proteins

To get more insights into the structural changes that may affect the UGT74S1 glycosyltransferase activity *in vitro*, a secondary dimension (2D) protein structural analysis of the mutant and the wild type proteins was performed. Mutations induced changes in the number of α-helices and β-strands and a high diversity in the β-strand number (S6 Fig.; S2 Table). The mutant Ser357Ala protein carrying an alanine mutation and the wild type showed the same number of α-helices and β-strands. In contrast, whereas all other mutants displayed 17 α-helices, the mutant protein Trp355Ala showed 16 α-helices. To further assess whether these structural changes affected the protein conformations and binding features, 3D molecular models of wild type and mutant of UGT74S1 were built (S7 Fig.) and their homology-based modeling matched the same UGT template with 100% confidence. The template and targets showed 24–28% identity, and overall, 458–463 amino acid residues were successfully modeled with more than 90% accuracy (S3 Table).

### Molecular docking of the six UGT74S1 mutants with UDP-glucose and SECO

To further assess whether site-directed mutagenesis affected the predicted UGT74S1 binding characteristics, a molecular modeling and docking study was conducted using each mutant variant as performed for the wild type UGT74S1. Gains, losses, or changes of amino acid binding sites were observed for all the six UGT74S1 mutant variants for UDP-glucose and SECO (Table 2, 3).

**Table 2:** UGT74S1 and its derived mutant binding positions to UDP-glucose as predicted by molecular docking.

Protein	Ligand	ZINC code	Number of hydrogen bonds	Amino acid binding Position	Binding functional group in ligand†	Bond size (Å)
UGT74S1	UDP-Glucose	30320665	2	Ser357	O8	2.207
				Trp355	O13	2.145
Cys335Ala	UDP-Glucose	30320665	2	Ser357	O8	2.216
				Asn356	O6	1.944
Gln337Ala	UDP-Glucose	30320665	3	Trp355	O6	2.226
				Ser283	O14	2.198
				Leu284	O14	2.23
Ser357Ala	UDP-Glucose	30320665	1	Trp355	O13	2.087
Trp355Ala	UDP-Glucose	30320665	1	Ser115	H17	2.117
Trp355Gly	UDP-Glucose	30320665	1	Gly355	O13	2.061
His352Asp	UDP-Glucose	30320665	4	Gly344	O8	2.015
				Ala342	H1	2.13
				Tyr263	O5	2.322
				Asn258	H21	2.650

Amino acids within the proteins and atoms of UDP-glucose involved in binding, and hydrogen bond length are shown.

† Atoms were numbered following the UCSF Chimera numbering system (which is different from standard chemistry numbering system).

**Table 3:** UGT74S1 and its derived mutant binding positions to SECO as predicted by molecular docking.

Protein	Ligand	ZINCcode	Number ofhydrogen bonds	Amino acidbinding Position	Binding functionalgroup in ligand†	Bond size(Å)
UGT74S1	SECO	14694366	4	Cys335	O2	2.566
				Cys335	H9	1.802
				Gln337	O1	2.512
				Trp355	O4	2.486
Cys335Ala	SECO	14694366	2	Asn356	O4	2.534
				Asn356	O5	2.588
Gln337Ala	SECO	14694366	2	Asn356	O4	2.426
				Asn356	O5	2.597
Ser357Ala	SECO	14694366	3	Cys335	O2	2.345
				Cys335	H9	1.895
				Gln337	O1	2.487
Trp355Ala	SECO	14694366	1	Ser180	H23	2.331
Trp355Gln	SECO	14694366	3	Cys335	O2	2.256
				Cys335	H9	1.829
				Gln337	O1	2.563
His352Asp	SECO	14694366	3	Cys335	O2	2.412
				Cys335	H9	1.923
				Gln337	O1	2.608

Amino acids within the proteins and atoms of SECO involved in binding, and hydrogen bond length are shown.

† Atoms were numbered following the UCSF Chimera numbering system (which is different from standard chemistry numbering system).

Using UDP-glucose as a ligand, two hydrogen bonds were found to be involved in binding UDP-glucose to the mutant Cys335Ala protein (Table 2; S8 Fig.). The first bond occurred between the oxygen atom 8 on phosphate group 2 of UDP-glucose, forming a 2.216 Å hydrogen bond with the serine at position 357 (Ser^357^). The second bond occurred between the oxygen atom 6 on phosphate group 1 of UDP-glucose, forming a 1.944 Å hydrogen bond with the asparagine at position 356 (Asn^356^). UDP-glucose was thus found to bind within the HCGWNS region of PSPG as observed with the wild type UGT74S1 protein. Contrary to the wild type, three hydrogen bonds were found to be involved in binding UDP-glucose to the mutant Gln337Ala. The first bond occurred between the oxygen atom 6 of phosphate group 1 of UDP-glucose, forming a 2.226 Å hydrogen bond with the tryptophan at position 355 (Trp^355^). The second and third bonds occurred between the oxygen atom 14 of the hydroxyl group located at position 12 of UDP-glucose, forming a 2.198 Å hydrogen bond with the serine 283 (Ser^283^) and a 2.230 Å hydrogen bond with the leucine 284 (Leu^284^), respectively. Unlike the wild type protein, the last two UDP-glucose binding sites (Ser^283^ and Leu^284^) appeared to be located outside the PSPG active site of the enzyme. A single hydrogen bond was predicted between UDP-glucose and each of the three mutant proteins Ser357Ala, Trp355Ala and Trp355Gly. In the interaction between the mutant protein Ser357Ala and UDP-glucose, the hydrogen bond occurred between the oxygen 13 of the hydroxyl group located at 13 position of UDP-Glucose, forming a 2.087 Å hydrogen bond with the tryptophan 355 (Trp^355^). This bond was identical to that formed by the wild type protein, but the substitution of Ser^357^ by Ala^357^ resulted in the loss of the second hydrogen bond with UDP glucose. With mutant protein Trp355Ala, the hydrogen bond occurred between the hydrogen 17 of the hydroxyl group located at position 15 of the UDP-Glucose moiety, forming a 2.117 Å hydrogen bond with the serine 115 (Ser^115^). The UDP-glucose binding site to the mutant protein Trp355Ala was therefore found to be located outside the PSPG active site of the enzyme. The Trp355Gly protein–UDP-glucose ligand interaction occurred through a hydrogen bond between the oxygen atom 13 of the hydroxyl group located at 13 position of UDP-Glucose and formed a 2.061 Å hydrogen bond with the glycine 355 (Gly^355^). The substitution of Trp^355^ by Gly^355^ did not change the binding at position 355 of the protein but resulted in the loss of the second hydrogen bond with UDP glucose. By substituting His^352^ to Asp^352^ in mutant His352Asp, four new binding sites between His352Asp protein and UDP-glucose were observed and involved amino acid residues threonine 263 (Thr^263^), asparagine 258 (Asn^258^), Ala^342^, and Gly^344^ (Table 2; S8 Fig.).

Using the sugar acceptor SECO as a ligand, the docking of each Cys335Ala and Gln337Ala mutant proteins predicted asparagine 356 (Asn^356^) forming two hydrogen bonds with SECO (Table 3; S8 Fig.). With both Cys335Ala and Gln337Ala mutant proteins, the interactions occurred between an Asn^356^ residue and oxygen atom 4 of the methoxy-ester group located at positions 17, and oxygen atom 5 of the hydroxyl group at position 16 of SECO (Table 3; S8 Fig.). Similar to Cys335Ala and Gln337Ala, the three mutant Ser357Ala, Trp355Gly and His352Asp proteins were predicted to bond SECO by three hydrogen bonds involving Cys^335^ and Gln^337^ amino acid residues and the oxygen atom 2 of hydroxyl group at position 7, the hydrogen 9 of hydroxyl group at position 7, and the oxygen 1 of the methoxy-ester group at position 2 of SECO. The mutant protein Trp355Ala was predicted to interact with SECO through a single bond involving Ser^180^ residue and the hydrogen 23 of the hydroxyl group at position 16 of SECO. The bond lengths between the three proteins and SECO were fairly similar (Table 3; S8 Fig.). None of the hydroxyl groups at positions 3 and 6; potential targets for glucosylation were involved in hydrogen bond formation with the proteins (S8 Fig.).

### In vitro heterologous protein expression and enzyme activity

**Figure 2:**
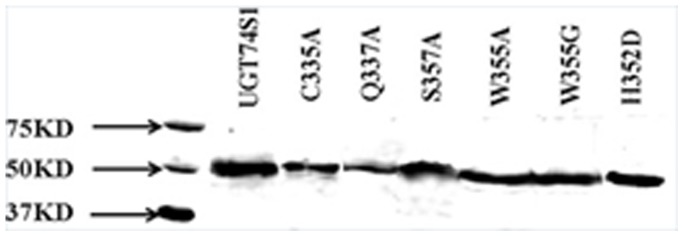
Western blot of HisTag-purified proteins from the wild type and six mutant UGT74S1 protein variants probed with antiXpress antibody. Mutant proteins are indicated by their one-letter amino acid codes. W, Tryp; C, Cys; Q, Gln; A, Ala; S, Ser; H, His; D, Asp. M, Western C precision plus protein marker mixed with conjugant (BioRad).

To assess the expression and functionality of the different mutant proteins, the full length cDNAs for the wild type *UGT74S1* and that of each of the 6 *UGT74S1* mutants were expressed *in vitro* in yeast. Similar to the wild type UGT74S1, all six mutants produced, along with the Histidine-Tag, a discrete protein band of 56.4 kDa (Fig. 2).

**Figure 3:**
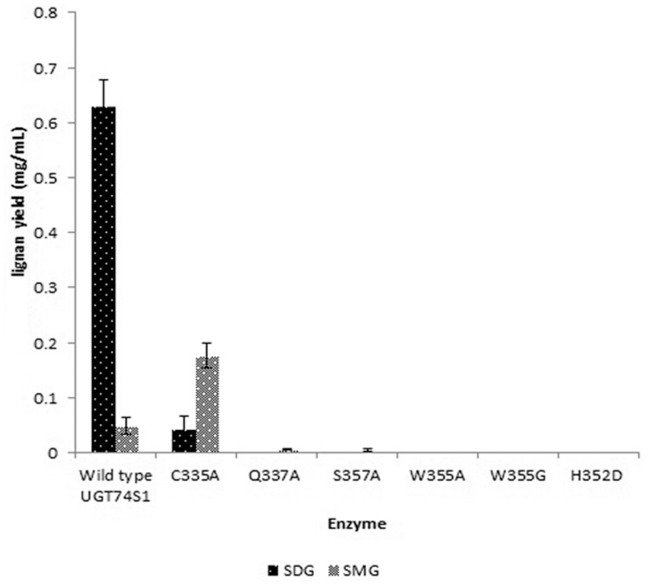
Comparative metabolite profiles obtained from enzyme assays with the wild type and the mutant UGT74S1 proteins using SECO as acceptor and UDP-glucose as sugar donor. SDG and SMG lignan yield (mg/mL) were obtained from three independent replicates. Vertical bars are standard deviations of the means. Mutant proteins are indicated by their one-letter amino acid codes. W, Tryp; C, Cys; Q, Gln; A, Ala; S, Ser; H, His; D, Asp.

To determine the biological effects of the predicted ligand binding site alterations induced by site-directed mutagenesis, enzyme assays were performed using the purified proteins from the wild type UGT74S1 and each of the 6 six mutant versions. A significant reduction of UGT74S1 glucosyltransferase activity towards SECO was observed in all mutants when compared to the wild type (Fig. 3). None of the UGT74S1 mutants or wild type were shown to glucosylate any of the other aglycone substrates tested, and only the substrate’s peaks were observed on chromatograms (data not shown). When Trp^355^ was substituted by either Ala or Gly, a complete abolition of activity was observed. No glucosylation activity was also observed when His^352^ was substituted by Asp^352^. Mutation of Cys^335^, Gln^337^ and Ser^357^ altered the lignan profiles in the reactions, with a significant reduction to null level of SDG production, while still producing SMG intermediate. Mutant *Cys335Ala* produced a significantly (P<0.001) lower level of SDG compared to the wild type, but produced a significantly higher (P<0.001) amount of SMG. Mutants *Gln337Ala* and *Ser357Ala* produced only SMG which was significantly (P<0.001) less than that produced by the wild type. No SDG was produced by these two mutants (Fig. 3).

### Wild and mutant UGT74S1 kinetic parameters

Under the optimal conditions (80 µg enzyme, 1 mM NaCl, pH 8.0, 30°C), the estimated apparent *Km* of the wild type and mutants (Cys335Ala and Gln337Ala) UGT74S1 proteins toward SECO or UDP-glucose were determined for SMG production (Table 4). The UGT74S1 mutants showed lower affinity (higher *Km*) for SECO and UDP-glucose compared to the wild type. The wild type UGT74S1 had a higher catalytic efficiency *(Kcat/Km*), reacting 10 and 112 times faster with SECO than Cys335Ala and Gln337Ala, respectively, and 1.64 and 6 times faster with UDP-glucose than the same mutants, respectively. These kinetic parameters clearly demonstrate that the UGT74S1 mutants were less efficient in converting the substrate into overall SMG products.

**Table 4:** Kinetic parameters of wild type and mutant UGT74S1 toward SECO and UDP-glucose.

Enzyme/ligand	*Km* (mM)	*Kca*t (sec^−1^)	*kcat/km* (min^−1^ mM^−1^)	*kcat/km* (sec^−1^mM^−1^)	Relative change
W/SECO	0.717	0.2303	19.269	0.3212	1
Cys335Ala/SECO	1.538	0.0494	1.927	0.0321	9.999
Gln337Ala/SECO	2.716	0.0077	0.171	0.0028	112.492
W/UDP-gluc	1.103	0.1351	7.340	0.1224	1
Cys335Ala/UDP-gluc	3.443	0.2562	4.460	0.0744	1.645
Gln337Ala/UDP-gluc	27.610	0.5473	1.189	0.0198	6.175

W, wild type UGT74S1; UDP-gluc, UDP-glucose.

## Discussions

Prediction of protein-ligand interactions has paved the way to rational amino acid residue mutagenesis as an approach for a better understanding of their biological roles in protein activity and catalysis [46]–[49]. In this study, molecular docking, site-directed mutagenesis, and enzyme activity assays were conducted to determine the role played by five amino acid residues located within the PSPG motif of the recently characterized flax UGT74S1 [29]. The 3D structure of the UGT47S1 protein was constructed and its binding sites to ligands were predicted. Assessment of the wild type and mutant proteins’ activities experimentally substantiated these predictions. Mutation of Trp^355^ and His^352^ completely abolished UGT74S1 enzyme activity toward SECO. Although, HCGWNS motif appears to be essential for UGT activity, binding to substrates, and product formation (based on the works from several other groups), this study is the first to report on a 2 step glucosylation enzyme and raised the possibility of producing a non-naturally occurring metabolite following mutagenesis within the motif. The findings not only confirmed our previous reports [29], but provided the first evidence that Trp^355^ and His^352^ are critical for UGT74S1 glucosylation activity toward SECO.

Using Phyre homology-based modeling, a 3D structure for UGT74S1 was produced with 90% accuracy and 100% confidence. The identity of UGT74S1 to known proteins was 25% and permitted an accurate modeling. Indeed, despite low primary sequence similarity, the secondary and tertiary structures of GTs are highly conserved [18], and less than 20% identity are commonly used for homology modeling by exploiting the 2D and 3D structure conservation between a query protein and a template protein of known structure [18], [32]. By docking the UGT74S1’s 3D structure with ligands, UDP-glucose and SECO were fitted in a pocket located in the PSPG region. UDP-glucose binds to Trp^355^ and Ser^357^ at the C-terminal portion of the PSPG, while SECO binds primarily to Cys^335^ and Gln^337^ at the N-terminal portion of this conserved motif, and one hydrogen bond established with Trp^355^ at the C-terminal region, consistent with previous reports [18], [35], [50]. These predicted binding sites served for rational targeted mutagenesis that produced six UGT74S1 mutants, unprecedented in flax.

Site-directed mutagenesis modified the protein secondary structure and binding sites to ligands. The wild UGT74S1 consisted of 17 α-helices and 13 β-strands. In contrast, a slightly higher number (14–15) of β-strands were observed in all mutants, except for mutant Ser357Ala which was made of 13 β-strands, as was the wild type UGT74S1. All mutants displayed 17 α-helices except the mutant Trp355Ala which was made of 16 α-helices and 15 β-strands, thus being different from the wild type and all other mutants. Hence, wild UGT74S1 and its mutant variants displayed only a slight 2D structure variation. Previous studies have described 14 α-helices and 12 β-strands in UGT85B1 [38] whereas 16 α-helices and 13 β-strands were reported in UGT94B1 [36]. These observations are in agreement with the concept that 2D and 3D structures of query proteins and templates of known structures are conserved despite variations in the primary structures [18], [32], [41] and are further indications for the accuracy of our modeling. Changes in the 2D protein structures after site-directed mutagenesis were also followed by changes in the predicted ligand binding site to UGT74S1 variants, although their protein expression patterns in yeast were not different. Alterations of the predicted ligand binding sites in mutants were substantiated by enzyme assays. Reduced SECO glucosylation activity and alterations in the amounts and type of end products were observed in mutants carrying Cys335Ala, Gln337Ala, and Ser357Ala proteins as evidenced by their lower catalytic efficiency (*Kcat/Km*) when compared with the wild type. Mutant Cys335Ala produced both SDG and SMG, with the latter being synthetized more than 3X higher than SDG. Mutants Gln337Ala and Ser357Ala produced only SMG, albeit in small amounts. Both mutant proteins were predicted to bind UDP-glucose at Trp^355^, suggesting its important role in SMG production. As to why only SMG was observed in 2 mutants, this may be attributable to their extreme low catalytic efficiency toward both SECO and UDP-glucose and as consequence, unable to achieve the second glucosylation step into SDG. Some UGTs such as UGT78D2 and UGT71C2 have been reported to glycosylate only the 3-OH or 7-OH positions, respectively, whereas other UGTs such as UGT88A1 were capable of recognizing multiple positions [31]. UGT74S1 is part of the latter group as reported by Ghose et al. [29] and mutation of its Gln^337^ and Ser^357^ led to glucosylation of a single position as shown in mutants Gln337Ala and Ser357Ala. Hence, Gln^337^ and Ser^357^can be engineered and used to produce a single metabolite (SMG) *in vitro* as this metabolite may be more bioavailable than SDG because of inter-individual differences in the gut microflora ensuring the deglucosylation of polyphenolics prior to absorption. The substitution of Trp^355^ by the hydrophobic amino acids Ala or Gly, and His^352^ by the negatively charged Asp resulted in the complete abolition of enzyme activity. It seems that these three mutations induced unsuitable UDP-glucose binding positions to the proteins and thus prevented the catalysis. This assumption is in line with the extreme low catalytic efficiency observed with the mutants Gln337Ala and Ser357Ala showing a much reduced activity. It is well known that a prerequisite and crucial point for activity is the position of the acceptor –OH, –NH_2_ or –SH functional groups amenable to glycosylation [18]. For UGTs, the accepting functional group needs to be positioned near the 1′C (C10 and C20 in this study; (S8 Fig.)) of the sugar-donor glucose and near to the amino acid that acts as a general base to facilitate the deprotonation of the acceptor. In most plant UGTs, this deprotonation amino acid is a histidine residue [40], [43]–[51], [52]. In this study, mutation of His^352^ located in the PSPG abolished UGT74S1 activity. Thus, it is reasonable to assume that His^352^ of UGT74S1 may be responsible for the deprotonation process of the acceptor SECO and its mutation to Asp^352^ impaired this process, despite UDP-glucose and SECO being positioned in the pocket along the PSPG motif. Mutation of Trp^355^ also abolished UGT74S1 activity. This amino acid residue interacted with both SECO and UDP-glucose in the wild type UGT74S1, and these interactions were altered in the mutants Trp355Ala and Trp355Gly and no activity occurred, providing evidence of its critical role in ligand binding and catalysis. Wild type UGT74S1 and three of its mutant versions showed glucosylation activity toward SECO only, among the aglycone substrates tested, suggesting exclusive SECO substrate specificity for this enzyme, and that His^352^ and Trp^355^ likely control the regiospecificity/regioselectivity of UGT74S1 for SECO glucosylation in flax.

## Conclusion

Flax UGT74S1 sequentially glucosylates SECO into SMG and SDG as previously reported [29] and for the first time, we provided convincing evidence that Gln^337^ and Ser^357^ are crucial for conversion of SMG to SDG by this enzyme and that Trp^355^ and His^352^ are essential for UGT74S1 glucosylation activity toward SECO. Since SMG is not accumulated *in planta*, is not available commercially, and two of the mutants described in this study produced only this metabolite, we believe that tools and resources are now available to produce SMG in a fermentation setting.

## Supporting Information

S1 Fig **UGT74S1 sequence used for designing the site-directed mutagenesis primers.** The regions covered by primer locations are underlined. The target-mutated nucleotides are shown in italic and bold characters and their corresponding amino acids are italicized and bold.
(TIF)

S2 Fig **3D structure of wild type UGT74S1 as obtained by Phyre.** The α-helix, β-strand, and coils are colored in red, yellow, and green, respectively. The PSPG region is indicated in blue.
(TIF)

S3 Fig **Secondary structure of wild type UGT74S1 as predicted by Phyre2 Investigator.** PSPG motif is indicated by a two-head red arrow; amino acid residues predicted within the enzyme pocket are colored in red. The mutational sensitivity of individual amino acid residues is colour-coded based on the sensitivity scale shown at the bottom of the figure. The symbols (green symbol) and (blue arrow) represent α-helix and β-strand, respectively.
(TIF)

S4 Fig **Graph presenting the predicted effect of amino acid residue mutations as generated by SusPect server analysis using UGT74S1 as template.** The 20 possible amino acid types are labelled along the x-axis with their one-letter code. The coloured bars indicate level of probability at which a mutation of the corresponding residue will have an effect on UGT74S1 protein function. The amino acids alanine, glycine and aspartic acid selected for replacing the five targeted amino acids are marked with a star symbol under their respective panel. The probability color code scale is presented at the bottom of the figure.
(TIF)

S5 Fig **ClustalW alignment of the wild type and the six mutant variants of UGT74S1.** Mutant proteins are named using their one-letter amino acid codes. W, Tryp; C, Cys; Q, Gln; A, Ala; S, Ser; H, His; D, Asp. The 5 targeted amino acids changed by site-directed mutagenesis are shown in each mutant.
(TIF)

S6 Fig **Comparative secondary structures of the wild type and the six mutant variants of UGT74S1.** Mutant proteins are named using their one-letter amino acid codes. W, Tryp; C, Cys; Q, Gln; A, Ala; S, Ser; H, His; D, Asp. The symbols (green symbol) and (blue arrow) represent α-helix and β-strand, respectively. The α-helix and β-strands are numbered from the N terminal to C terminal end of the proteins.
(TIF)

S7 Fig **Comparative 3D modelling of the wild type and the six mutant variants of UGT74S1 as generated by Phyre2 Server.** Mutant proteins are named using their one-letter amino acid codes. W, Tryp; C, Cys; Q, Gln; A, Ala; S, Ser; H, His; D, Asp. The coordinates used to generate each structure is indicated below the given structure. The α-helix, β-strand, and coils are colored in red, yellow and green, respectively. The PSPG region is indicated in blue; amino acids targeted for site-directed mutagenesis are shown in pink. The mutated amino acids in mutants are shown in cyan blue.
(TIF)

S8 Fig **Comparative molecular docking of the wild type UGT74S1 and the six mutants using SECO and UDP glucose as ligands.** The structure of ligand SECO and UDP-glucose are presented on top of the docking models where atoms are numbered following the UCSF Chimera numbering system. Mutant proteins are named using their one-letter amino acid codes. W, Tryp; C, Cys; Q, Gln; A, Ala; S, Ser; H, His; D, Asp. Only the portion of the protein interacting with ligands is shown. α-helix, β-strand, and coil are colored in red, yellow green, respectively; the hydrogen bonds between amino acids and ligands and their respective length (Å) are indicated in black. The PSPG region is indicated in blue; amino acid residues involved in binding the sugar donor UDP-glucose or sugar acceptor SECO are colored in pink; The mutated amino acids are shown in cyan blue; the ligand oxygen atoms involved in hydrogen bond formation are circled in orange; the ligand hydrogen atoms involved in hydrogen bond formation are circled in grey; the SECO ligand oxygen atoms targeted for glucosylation are circled in blue.
(TIF)

S1 Table **Forward and Reverse Primers used for site-directed mutagenesis of UGT74S1.**
(DOCX)

S2 Table **Description of α-helices and β-strands found in wild type and mutants of UGT74S1.** The number of α-helices and β-strands, and percentage of amino acids involved in the α-helices, β-strands as well number of disordered structures are shown.
(DOCX)

S3 Table **Details of template protein (PDB code, confidence, identity) matching with the query sequence and used for docking the wild type Lu-UGT74S1 and the six mutant proteins.**
(DOCX)

## References

[1] Dixon RA (2004) Phytoestrogens. Annu Rev Plant Biol 55:225–61. 15377220 10.1146/annurev.arplant.55.031903.141729

[2] Thompson LU, Boucher BA, Liu Z, Cotterchio M, Kreiger N (2006) Phytoestrogen content of foods consumed in Canada including isoflavones lignans and coumestan Nutr Cancer. 54:184–201. 10.1207/s15327914nc5402_5 16898863

[3] Arroo RRJ, Androutsopoulos V, Beresford K, Ruparelia K, Surichan S, et al (2009) Phytoestrogens as natural prodrugs in cancer prevention: dietary flavonoids. Phytochem Rev 8:375–386.

[4] Prakash D, Gupta C (2011) Role of Phytoestrogens as Nutraceuticals in Human Health. Pharmacologyonline 1:510–523.

[5] Rabetafika HN, Remoortel VV, Danthine S, Paquot M, Blecker C (2011) Flaxseed proteins: food uses and health benefits. Intern J Food Sci Tech 46:221–228.

[6] Buck K, Zaineddin AK, Vrieling A, Heinz J, Linseisen J, et al (2011) Estimated enterolignans lignan-rich foods and fibre in relation to survival after postmenopausal breast cancer. British J Cancer 105:1151–1157. 10.1038/bjc.2011.374 PMC3208499 21915130

[7] Webb AL, McCullough ML (2005) Dietary lignans: potential role in cancer prevention. Nutrition and Cancer 51:117–131. 15860433 10.1207/s15327914nc5102_1

[8] Adolphe JL, Whiting SJ, Juurlink BHJ, Thorpe LU, Alcorn J (2010) Health effects with consumption of the flax lignan secoisolariciresinol diglucoside. British J Nutrition 103:929–938. 10.1017/S0007114509992753 20003621

[9] Pan JY, Chen SL, Yang MH, Wu J, Sinkkonen J, et al (2009) An update on lignans: natural products and synthesis Nat Prod Rep. 26:1251–1292. 10.1039/b910940d 19779640

[10] Noguchi A, Fukui Y, Iuchi-Okada A, Kakutani S, Satake H, et al (2008) Sequential glucosylation of furofuran lignan (+)-sesaminol by *Sesamum indicum* UGT71A9 and UGT94D1 glucosyltransferase. Plant J 54:415–427. 18248594 10.1111/j.1365-313X.2008.03428.x

[11] Yousefzadi M, Sharif M, Behmanesh M, Moyano E, Bonfill M, et al (2010) Podophyllotoxin: Current approaches to its biotechnological production and future challenges. Eng Life Sci 10:281–292.

[12] Berim A, Ebel R, Schneider B, Petersen M (2008) UDP-glucose:(6-methoxy) podophyllotoxin 7-*O*-glucosyltransferase from suspension cultures of *Linum nodiflorum* . Phytochem 69:374–381. 10.1016/j.phytochem.2007.07.030 17870138

[13] Ono E, Kim HJ, Murata J, Morimoto K, Okazawa A, et al (2010) Molecular and functional characterization of novel furofuran-class lignan glucosyltransferase from Forsythia. Plant Biotech 27:317–324.

[14] Jhala AM, Hall LM (2010) Flax (*Linum usitatissimum* L): Current Uses and Future Applications. Australian J Basic and Applied Sci 4:4304–4312.

[15] Touré A, Xueming X (2010) Flaxseed lignans: Source biosynthesis metabolism antioxidant activity Bio-active components and health benefits. Comprehensive Rev Food Sci and Food Safety 9:261–269. 10.1111/j.1541-4337.2009.00105.x 33467817

[16] During A, Debouche C, Raas T, Larondelle Y (2012) Among Plant Lignans Pinoresinol Has the Strongest Anti-inflammatory Properties in Human Intestinal Caco-2 Cells. Journal of Nutrition 142:1798–1805. 22955517 10.3945/jn.112.162453

[17] Gachon CM, Langlois-Meurinne M, Saindrenan P (2005) Plant secondary metabolism glycosyltransferases: the emerging functional analysis. Trends Plant Sci 10:542–549. 16214386 10.1016/j.tplants.2005.09.007

[18] Osmani SA, Bak S, Møller BL (2009) Substrate specificity of plant UDP-dependent glycosyltransferases predicted from crystal structures and homology modeling. Phytochem 70:325–347. 10.1016/j.phytochem.2008.12.009 19217634

[19] Bowles D, Isayenkova J, Lim E (2005) Poppenberger B Glycosyltransferases: managers of small molecules. Curr Opin Plant Biol 8:254–263. 15860422 10.1016/j.pbi.2005.03.007

[20] Bowles D, Lim E-K (2010) Glycosyltransferases of Small Molecules: Their Roles in Plant Biology. In: Encyclopedia of Life Sciences (ELS) John Wiley Sons Ltd: Chichester. DOI: 101002/9780470015902a0021537.

[21] Wang J, Hou B (2009) Glycosyltransferases: key players involved in the modification of plant secondary metabolites. Front Biol 4:39–46.

[22] Geisler-Lee J, Geisler M, Coutinho PM, Segerman B, Nishikubo N, et al (2006) Poplar carbohydrate-active enzymes Gene identification and expression analyses. Plant Physiol 140:946–962. 16415215 10.1104/pp.105.072652 PMC1400564

[23] Yonekura-Sakakibara K, Hanada K (2011) An evolutionary view of functional diversity in family 1 glycosyltransferases. Plant J 66:182–193. 21443631 10.1111/j.1365-313X.2011.04493.x

[24] Caputi L, Malnoy M, Goremykin V, Nikiforova S, Martens S (2012) A genome-wide phylogenetic reconstruction of family 1 UDP-glycosyltransferases revealed the expansion of the family during the adaptation of plants to life on land. Plant J 69:1030–1042. 22077743 10.1111/j.1365-313X.2011.04853.x

[25] Ross J, Li Y, Lim EK, Bowles DJ (2001) Higher plant glycosyltransferases. Genome Biol 2:30041–30046. 10.1186/gb-2001-2-2-reviews3004 PMC138907 11182895

[26] Witte S, Moco S, Vervoort J, Matern U, Martens S (2009) Recombinant expression and functional characterisation of regiospecific flavonoid glycosyltransferases from *Hieracium pilosella* L. Planta. 229:1135–1146. 10.1007/s00425-009-0902-x 19238428

[27] Barvkar VT, Pardeshi VC, Kale SM, Kadoo NY, Gupta VS (2012) Phylogenomic analysis of UDP glycosyltransferase 1 multigene family in *Linum usitatissimum* identified genes with varied expression patterns. BMC Genomics 13:175. doi:101186/1471-2164-13-175. 22568875 10.1186/1471-2164-13-175 PMC3412749

[28] Wang Z, Hobson N, Galindo L, Zhu S, Shi D, et al (2012) The genome of flax (*Linum usitatissimum*) assembled de novo from short shotgun sequence reads. Plant J 72:461–473. 22757964 10.1111/j.1365-313X.2012.05093.x

[29] Ghose K, Selvaraj K, McCallum J, Kirby CW, Sweeney-Nixon M, et al (2014) Identification and functional characterization of a flax UDP-glycosyltransferase glucosylating secoisolariciresinol (SECO) into secoisolariciresinol monoglucoside (SMG) and diglucoside (SDG). BMC Plant Biol 14:82. doi:101186/1471-2229-14-82. 24678929 10.1186/1471-2229-14-82 PMC3986616

[30] He XZ, Wang X, Dixon RA (2006) Mutational analysis of the *Medicago* glycosyltransferase UGT71G1 reveals residues that control regiospecificity for (Iso) flavonoid glycosylation. J Biol Chem 281:34441–34447. 16982612 10.1074/jbc.M605767200

[31] Cartwright AM, Lim E-K, Kleanthous C, Bowles DJ (2008) A kinetic analysis of regiospecific glucosylation by two glycosyltransferases of *Arabidopsis thaliana*: Domain swaping to introduce new activities. J Biol Chem 283:15724–15731. 18378673 10.1074/jbc.M801983200 PMC3259630

[32] Kelley L, Sternberg MJE (2009) Protein structure prediction on the web: a case study using the Phyre server. Nature Protocols 4:363–371. 19247286 10.1038/nprot.2009.2

[33] Grosdidier A, Zoete V, Michielin O (2007) EADock: Docking of small molecules into protein active sites with a multiobjective evolutionary optimization. Proteins 67:1010–1025. 17380512 10.1002/prot.21367

[34] Grosdidier A, Zoete V, Michielin O (2011) SwissDock a protein-small molecule docking web service based on EADock DSS. Nucleic Acids Research 39: W270–W277 Web Server issue. doi: 101093/nar/gkr366. 10.1093/nar/gkr366 PMC3125772 21624888

[35] Bhat WW, Dhar N, Rzdan S, Rana S, Mehra R, et al (2013) Molecular characterization of UGT94F2 and UGT86C4 two glycosyltranferase from *Picrorhiza kurrooa*: Comparative structural insight and evaluation of substrate recognition. PLOS One 8:e73804. 24066073 10.1371/journal.pone.0073804 PMC3774767

[36] Hiromoto T, Honjo E, Tamada T, Noda N, Kazuma K, et al (2013) Crystal structure of UGP-glucose:anthocyanidin 3-*O*-glucosyltransferase from *Clitoria ternatea* . J Synchrotron Rad 20:894–898. 10.1107/S0909049513020712 PMC3795551 24121335

[37] Imberty A, Wimmerova M, Koca J, Breton C (2006) Molecular modeling of glycosyltranferases. Methods Mol Biol 347:145–156. 17072009 10.1385/1-59745-167-3:145

[38] Thorsoe KS, Bak S, Olsen CE, Imberty A, Breton C, et al (2005) Determination of catalytic key amino acids and UDP-sugar donor specificity of the cyanohydrin glycosyltranferase UGT85B1 from *Sorghum bicolor*: molecular modeling substantiated by by site-specific mutagenesis and biochemical analysis. Plant Physiol 139:664–673. 16169969 10.1104/pp.105.063842 PMC1255986

[39] Osmani SA, Bak S, Imberty A, Olsen CE, Moller BL (2008) Catalytic key amino acids and UDP-sugar donor specificity of plant glucuronosyltransferase UGT94B1: Molecular modeling substantiated by site-specific mutagenesis and biochemical analyses. Plant Physiol 148:1295–1308. 18829982 10.1104/pp.108.128256 PMC2577238

[40] Shao H, He X, Achine L, Blout JW, Dixon RA, et al (2005) Crystal structures of multifunctional triterpene/flavoinoid glycosyltranferase from *Medicago truncatula* . Plant cell 17:3141–3154. 16214900 10.1105/tpc.105.035055 PMC1276034

[41] Modolo LV, Escamilla-Trevino, Dixon RA, Wang X (2009) Single amino acid mutations of *Medicago* glycosyltransferase UGT85H2 enhance activity and impart reversibility. Febs Letters 583:2131–2135. 19500551 10.1016/j.febslet.2009.05.046

[42] Noguchi A, Saito A, Homma Y, Nakao M, Sasaki N, et al (2007) A UDP-glucose:isoflavone 7-*O*-glucosyltransferase from the roots of soybean (*Glycine max*) seedlings Purification gene cloning phylogenetics and an implication for an alternative strategy of enzyme catalysis. J Biol Chem 282:23581–23590. 17565994 10.1074/jbc.M702651200

[43] Brazier-Hicks M, Offen WA, Gershater MC, Revett TJ, Lim EK, et al (2007) Characterization and engineering of the bifunctional *n*- and *o*-glucosyltransferase involved in xenobiotic metabolism in plants. Proc Nat Acad Sci USA 104:p20238. 10.1073/pnas.0706421104 PMC2154415 18077347

[44] Irwin JJ, Shoichet BK (2005) ZINC-a free database of commercially available compounds for virtual screening. J Chem Inf Model 45:177–182. 15667143 10.1021/ci049714 PMC1360656

[45] Pettersen EF, Goddard TD, Huang CC, Couch GS, Greenblatt DM, et al (2004) UCSF Chimera - a visualization system for exploratory research and analysis. J Comput Chem 25:1605–1612. 15264254 10.1002/jcc.20084

[46] Looger LL, Dwyer MA, Smith JJ, Hellinga HW (2003) Computational design of receptor and sensor proteins with novel functions. Nature 423:132–133. 12736688 10.1038/nature01556

[47] Cai J, Han C, Hu T, Zhang J, Wu D, et al (2006) Peptide deformylase is a potential target for anti-Helicobacter pylori drugs: reverse docking enzymatic assay and X-ray crystallography validation. Protein Sci 15:2071–2081. 16882991 10.1110/ps.062238406 PMC2242601

[48] Röthlisberger D, Khersonsky O, Wollacott AM, Jiang L, DeChancie J, et al (2008) Kemp elimination catalysts by computational enzyme design. Nature 453:190–195. 18354394 10.1038/nature06879

[49] Wits S, Panwar P, Schober M, Deppe J, Pasha FA, et al (2014) Structure-function relationship of a plant NCS1 member – homology modfeling and mutagenesis identified resifues critical for substrate specificity in PLUTO a nucleobase transporter from *Arabidopsis* . Plos One 9:e91343. 24621654 10.1371/journal.pone.0091343 PMC3951388

[50] Masada S, Terasaka K, Mizukami H (2007) A single amino acid in the PSPG-box plays an important role in the catalytic function of caUGT2 (Cucurmin glucosultranferase) a group d family glycosyltranferase 1 from *Catharantus roseus* . FEBS Lett 581:2605–2610. 17509574 10.1016/j.febslet.2007.05.002

[51] Li L, Modolo LV, Escamilla-Trevino LL, Achnine L, Dixon RA, et al (2007) Crystal structure of Medicago truncatula UGT85H2– insights into the structural basis of a multifunctional (Iso) flavonoid glycosyltransferase. J Mol Biol 370:951–963. 17553523 10.1016/j.jmb.2007.05.036

[52] Offen W, Martinez-Fleites C, Yang M, Kiat-Lim E, Davis BG, et al (2006) Structure of a flavonoid glucosyltransferase reveals the basis for plant natural product modification. EMBO J 25:1396–1405. 16482224 10.1038/sj.emboj.7600970 PMC1422153

