# Supplementary figures and images for: Histidine 352 (His352) and Tryptophan 355 (Trp355) Are Essential for Flax UGT74S1 Glucosylation Activity toward Secoisolariciresinol
Source: PLoS One. 2015 Feb 25;10(2):e116248. doi: 10.1371/journal.pone.0116248 (PMC4340967; doi:10.1371/journal.pone.0116248)

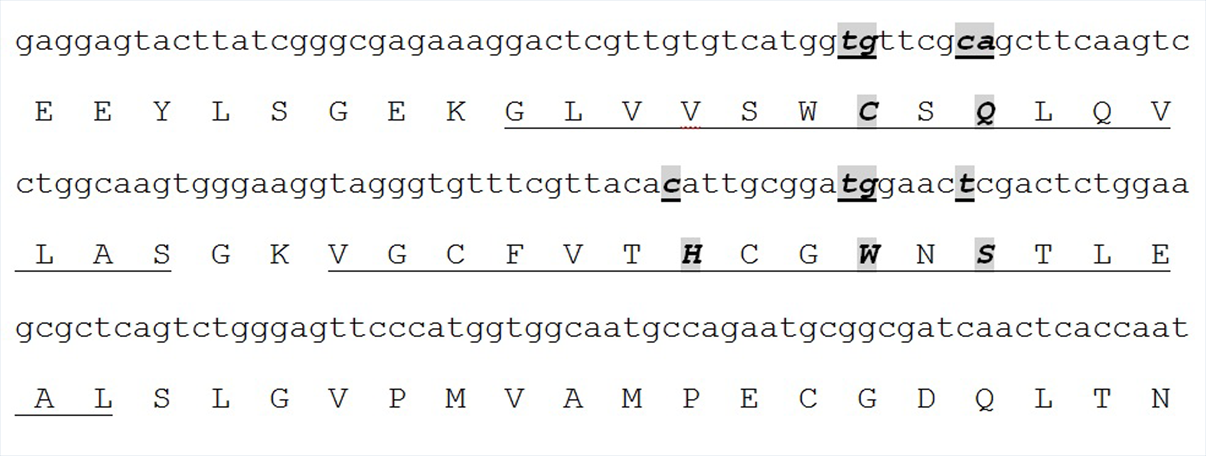

Supplement: S1 Fig — UGT74S1 sequence used for designing the site-directed mutagenesis primers. The regions covered by primer locations are underlined. The target-mutated nucleotides are shown in italic and bold characters and their corresponding amino acids are italicized and bold. (TIF) [file pone.0116248.s001.tif]

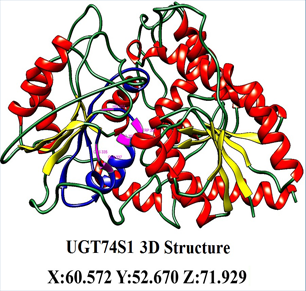

Supplement: S2 Fig — 3D structure of wild type UGT74S1 as obtained by Phyre. The α-helix, β-strand, and coils are colored in red, yellow, and green, respectively. The PSPG region is indicated in blue. (TIF) [file pone.0116248.s002.tif]

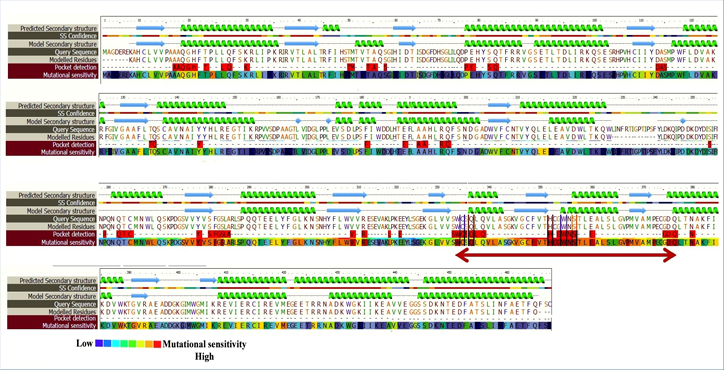

Supplement: S3 Fig — Secondary structure of wild type UGT74S1 as predicted by Phyre2 Investigator. PSPG motif is indicated by a two-head red arrow; amino acid residues predicted within the enzyme pocket are colored in red. The mutational sensitivity of individual amino acid residues is colour-coded based on the sensitivity scale shown at the bottom of the figure. The symbols (green symbol) and (blue arrow) represent α-helix and β-strand, respectively. (TIF) [file pone.0116248.s003.tif]

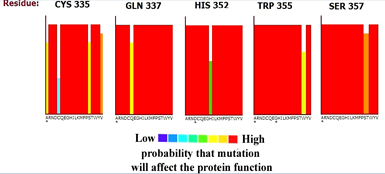

Supplement: S4 Fig — Graph presenting the predicted effect of amino acid residue mutations as generated by SusPect server analysis using UGT74S1 as template. The 20 possible amino acid types are labelled along the x-axis with their one-letter code. The coloured bars indicate level of probability at which a mutation of the corresponding residue will have an effect on UGT74S1 protein function. The amino acids alanine, glycine and aspartic acid selected for replacing the five targeted amino acids are marked with a star symbol under their respective panel. The probability color code scale is presented at the bottom of the figure. (TIF) [file pone.0116248.s004.tif]

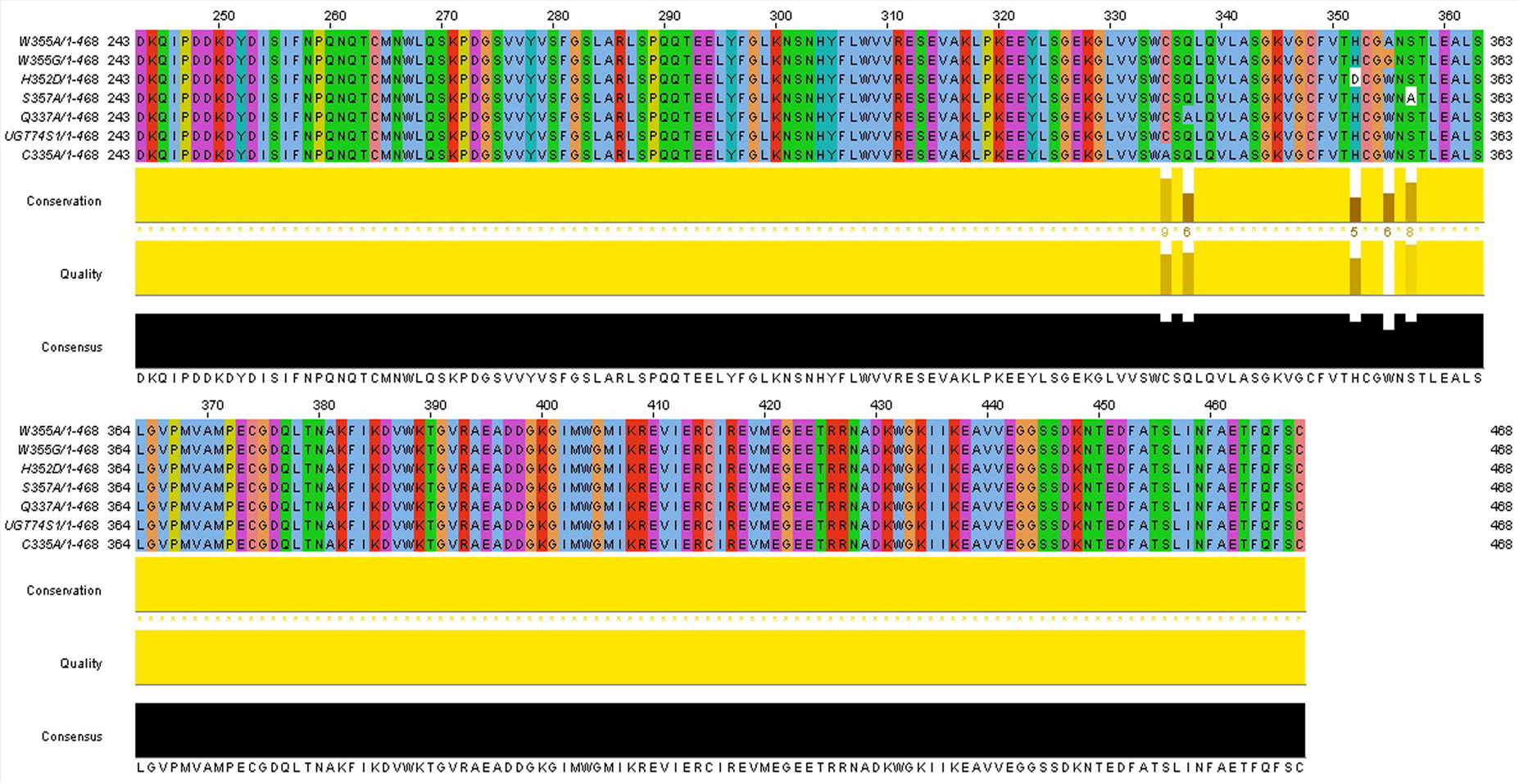

Supplement: S5 Fig — ClustalW alignment of the wild type and the six mutant variants of UGT74S1. Mutant proteins are named using their one-letter amino acid codes. W, Tryp; C, Cys; Q, Gln; A, Ala; S, Ser; H, His; D, Asp. The 5 targeted amino acids changed by site-directed mutagenesis are shown in each mutant. (TIF) [file pone.0116248.s005.tif]

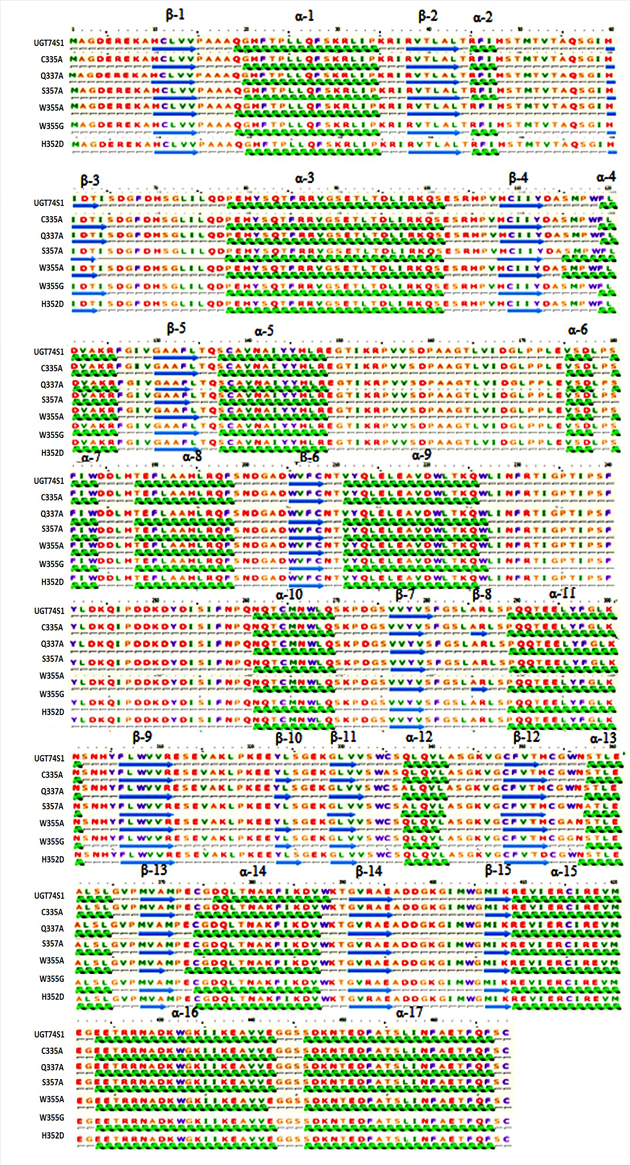

Supplement: S6 Fig — Comparative secondary structures of the wild type and the six mutant variants of UGT74S1. Mutant proteins are named using their one-letter amino acid codes. W, Tryp; C, Cys; Q, Gln; A, Ala; S, Ser; H, His; D, Asp. The symbols (green symbol) and (blue arrow) represent α-helix and β-strand, respectively. The α-helix and β-strands are numbered from the N terminal to C terminal end of the proteins. (TIF) [file pone.0116248.s006.tif]

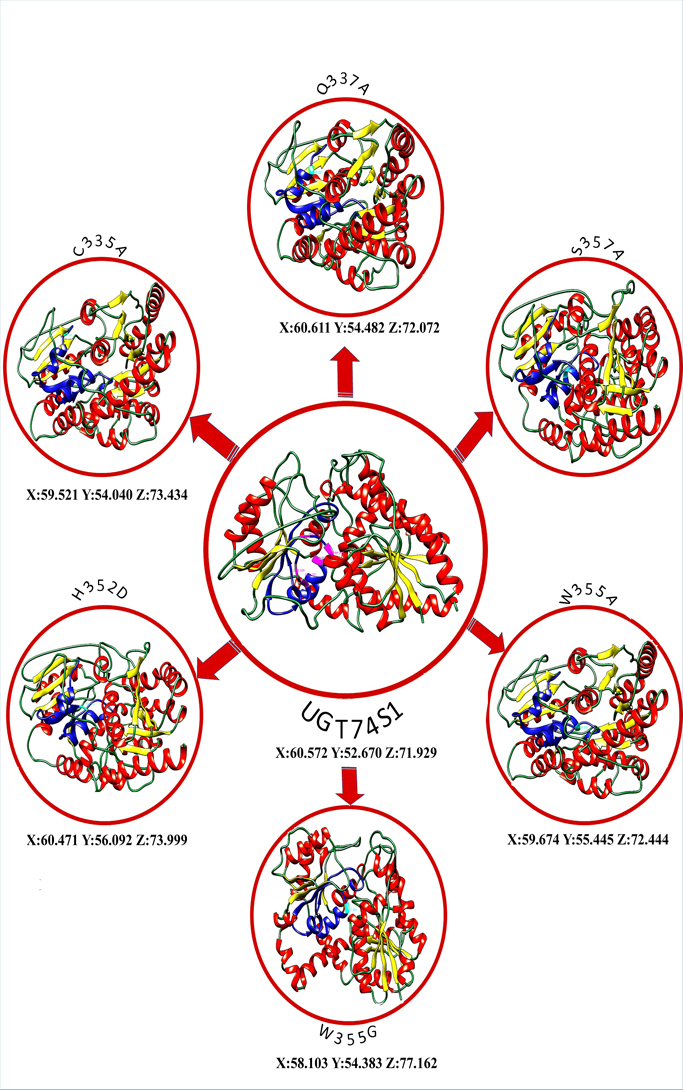

Supplement: S7 Fig — Comparative 3D modelling of the wild type and the six mutant variants of UGT74S1 as generated by Phyre2 Server. Mutant proteins are named using their one-letter amino acid codes. W, Tryp; C, Cys; Q, Gln; A, Ala; S, Ser; H, His; D, Asp. The coordinates used to generate each structure is indicated below the given structure. The α-helix, β-strand, and coils are colored in red, yellow and green, respectively. The PSPG region is indicated in blue; amino acids targeted for site-directed mutagenesis are shown in pink. The mutated amino acids in mutants are shown in cyan blue. (TIF) [file pone.0116248.s007.tif]

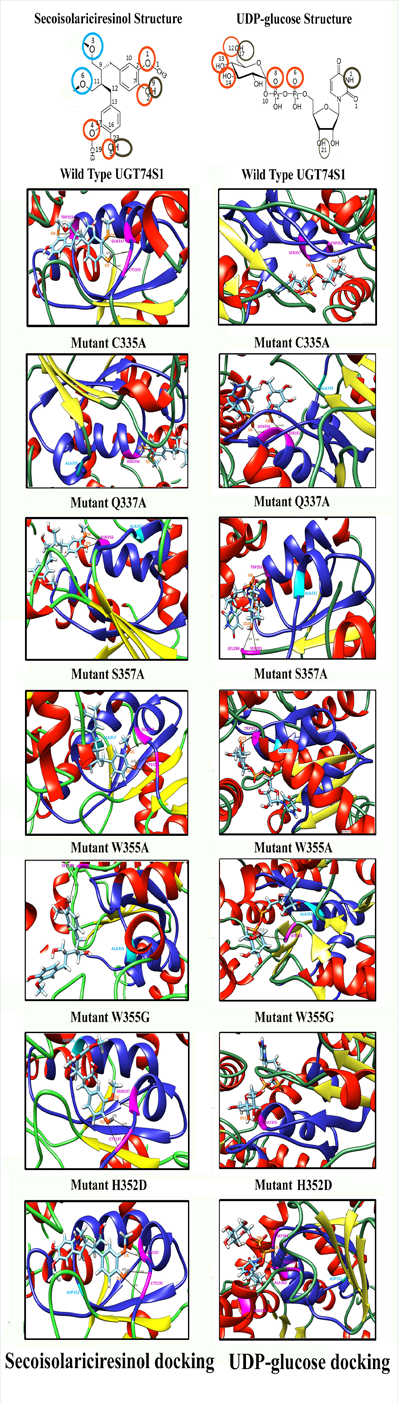

Supplement: S8 Fig — Comparative molecular docking of the wild type UGT74S1 and the six mutants using SECO and UDP glucose as ligands. The structure of ligand SECO and UDP-glucose are presented on top of the docking models where atoms are numbered following the UCSF Chimera numbering system. Mutant proteins are named using their one-letter amino acid codes. W, Tryp; C, Cys; Q, Gln; A, Ala; S, Ser; H, His; D, Asp. Only the portion of the protein interacting with ligands is shown. α-helix, β-strand, and coil are colored in red, yellow green, respectively; the hydrogen bonds between amino acids and ligands and their respective length (Å) are indicated in black. The PSPG region is indicated in blue; amino acid residues involved in binding the sugar donor UDP-glucose or sugar acceptor SECO are colored in pink; The mutated amino acids are shown in cyan blue; the ligand oxygen atoms involved in hydrogen bond formation are circled in orange; the ligand hydrogen atoms involved in hydrogen bond formation are circled in grey; the SECO ligand oxygen atoms targeted for glucosylation are circled in blue. (TIF) [file pone.0116248.s008.tif]
