# Supplementary material for: Histidine 352 (His352) and Tryptophan 355 (Trp355) Are Essential for Flax UGT74S1 Glucosylation Activity toward Secoisolariciresinol
Source: PLoS One. 2015 Feb 25;10(2):e116248. doi: 10.1371/journal.pone.0116248 (PMC4340967; doi:10.1371/journal.pone.0116248)
Supplement: S2 Table — Description of α-helices and β-strands found in wild type and mutants of UGT74S1. The number of α-helices and β-strands, and percentage of amino acids involved in the α-helices, β-strands as well number of disordered structures are shown. (DOCX) [file pone.0116248.s010.docx]

**Table S2.** Description of α-helices and β-strands found in wild type and mutants of UGT74S1. The number of α-helices and β-strands, and percentage of amino acids involved in the α-helices, β-strands as well number of disordered structures are shown.

| Protein name | α-helix | β-strand | percentage of amino acids involved | | |
| --- | --- | --- | --- | --- | --- |
|  |  |  | α-helix | β-strand | Disordered |
| UGT74S1 | 17 | 13 | 42 | 13 | 16 |
| Cys335Ala | 17 | 15 | 42 | 14 | 17 |
| Gln337Ala | 17 | 14 | 42 | 13 | 16 |
| Ser357Ala | 17 | 13 | 42 | 13 | 16 |
| Trp355Ala | 16 | 15 | 42 | 13 | 15 |
| Trp355Gly | 17 | 14 | 42 | 13 | 16 |
| His352Asp | 17 | 14 | 42 | 13 | 16 |

**Table S3**. Details of template protein (PDB code, confidence, identity) matching with the query sequence and used for docking the wild type Lu-UGT74S1 and the six mutant proteins.

| Protein name | Amino acid residues modelled | Template PDB  code | Confidence (%) | Resolution (Å) | identity (%) | Template Information |
| --- | --- | --- | --- | --- | --- | --- |
| UGT74S1 | 458 | d2vcha1 | 100.0 | 1.45 | 25 | hydroquinone glucosyltransferase **(**Q9M156**-1)** |
| Cys335Ala | 463 | d2vcha1 | 100.0 | 1.45 | 25 | hydroquinone glucosyltransferase **(**Q9M156**-1)** |
| Gln337Ala | 463 | d2vcha1 | 100.0 | 1.45 | 24 | hydroquinone glucosyltransferase **(**Q9M156**-1)** |
| Ser357Ala | 463 | d2vcha1 | 100.0 | 1.45 | 24 | hydroquinone glucosyltransferase **(**Q9M156**-1)** |
| Trp355Ala | 463 | d2vcha1 | 100.0 | 1.45 | 24 | hydroquinone glucosyltransferase **(**Q9M156**-1)** |
| Trp355Gly | 458 | d2vcha1 | 100.0 | 1.45 | 28 | hydroquinone glucosyltransferase **(**Q9M156**-1)** |
| His352Asp | 463 | d2vcha1 | 100.0 | 1.45 | 25 | hydroquinone glucosyltransferase **(**Q9M156**-1)** |
